# Correction: Detecting molecular interactions in live-cell single-molecule imaging with proximity-assisted photoactivation (PAPA)

**DOI:** 10.7554/eLife.97099

**Published:** 2024-02-20

**Authors:** Thomas GW Graham, John Joseph Ferrie, Gina M Dailey, Robert Tjian, Xavier Darzacq

**Keywords:** Human

Graham TGW, Ferrie JJ, Dailey GM, Tjian R, Darzacq X. 2022. Detecting molecular interactions in live-cell single-molecule imaging with proximity-assisted photoactivation (PAPA). *eLife* **11**:e76870. doi: 10.7554/eLife.76870.

Published 17 August 2022

After publication, we realized that due to mislabelling of equipment, we incorrectly reported the wavelength of our red laser as 633 nm, when in fact we used a Genesis MX639-1000 laser with a peak wavelength of 639 nm. This does not change the conclusions of our paper, but we apologize for the inaccuracy. All instances of ‘633 nm’ in the text and figures have been corrected to ‘639 nm’.

The Acknowledgements section was updated to include the following sentence:

The microscope used for fluorescence lifetime imaging was purchased with funding from NIH grant S10OD025063.

